# Correlations among improvements in urgency urinary incontinence, health-related quality of life, and perception of bladder-related problems in incontinent subjects with overactive bladder treated with tolterodine or placebo

**DOI:** 10.1186/1477-7525-7-13

**Published:** 2009-02-18

**Authors:** Philip EV Van Kerrebroeck, Con J Kelleher, Karin S Coyne, Zoe Kopp, Marina Brodsky, Joseph T Wang

**Affiliations:** 1 Department of Urology, University Hospital Maastricht, Maastricht, The Netherlands; 2 Guys and St. Thomas' Hospital NHS Trust, London, UK; 3 United Biosource Corporation, Center for Health Outcomes Research, Bethesda, MD, USA; 4 Pfizer Inc, New York, NY, USA

## Abstract

**Background:**

Previous studies demonstrate that tolterodine extended release (ER) significantly improves urgency urinary incontinence (UUI) episodes. Instruments that measure patient-reported outcomes (PROs) provide additional information that is valuable for assessing whether clinical improvements are meaningful to the patient. This study determined the correlation of changes in bladder diary variables and other PROs in subjects with overactive bladder (OAB).

**Methods:**

Subjects with OAB, urinary frequency, and UUI were treated with 4 mg once-daily tolterodine ER or placebo for 12 weeks. Subjects completed 7-day bladder diaries, the Patient Perception of Bladder Condition (PPBC), and the King's Health Questionnaire (KHQ) at baseline and week 12. Only subjects who reported at least some minor bladder-related problems at baseline (PPBC score ≥ 3) were included in this analysis.

**Results:**

Reductions in UUI episodes per week were significantly greater in the tolterodine ER group (n = 500) compared with the placebo group (n = 487) at week 12 (-71% vs -33%, *P *< 0.0001). A significantly greater percentage of subjects in the tolterodine ER group reported improvement on the PPBC versus placebo (58% vs 45%, *P *< 0.0001), and 7 of 10 KHQ domains were significantly improved versus placebo (all *P *< 0.05). Significant correlations were found for median percentage changes in UUI episodes with changes in PPBC scores (*r *= 0.35,*P *< 0.0001) and the 7 improved KHQ domains (*r *= 0.16–0.32, *P *≤ 0.0011). Changes in PPBC scores and all KHQ domains were significantly correlated (*r *= 0.13–0.38, *P *≤ 0.009) in the tolterodine ER group. Correlations among endpoints in the placebo group were similar to those observed in the tolterodine ER group.

**Conclusion:**

Improvement in UUI episodes after 12 weeks of treatment with tolterodine ER or placebo was correlated with improvements in patients' perception of their bladder-related problems and health-related quality of life. Correlations were moderate in magnitude but statistically significant, suggesting that PROs are important and relevant measures for evaluating OAB treatment.

## Background

Overactive bladder (OAB) is defined by the International Continence Society (ICS) as urgency, with or without urgency urinary incontinence (UUI), usually with increased daytime frequency and nocturia [1,2]. OAB affects approximately 17% of adults in the United States [3] and Europe [4]. A recent study using current ICS definitions of OAB found the prevalence of OAB to be 11% in men and 13% in women [5]. It was also demonstrated that the prevalence of OAB increases with advancing age, occurring in 20% of subjects who are ≥ 60 years of age. Antimuscarinics, such as tolterodine, are first-line treatments for OAB and have proven efficacy and tolerability [6].

Bladder diaries are traditionally used to quantify OAB symptoms and provide information about treatment effects on the frequency and severity of symptoms, but they do not provide information regarding the patient's perspective on his or her symptoms or the impact of treatment. Instruments that measure patient-reported outcomes (PROs) provide valuable additional information for assessing whether clinical improvements are meaningful to the patient [7]. Several questionnaires, measuring a broad array of concepts, have been validated for use in subjects with OAB, including the King's Health Questionnaire (KHQ) [8] and Patient Perception of Bladder Condition (PPBC) [9].

Numerous studies have used questionnaires to demonstrate that OAB symptoms have a negative impact on the subjects' health-related quality of life (HRQL) [3,10-13]. OAB adversely affects social and professional activities, social and sexual relationships, and travel [14,15]. Treatment with antimuscarinics is associated with significant improvement in HRQL, symptom bother, and perception of treatment benefit, as well as improvement in bladder diary variables [16-19]. Evidence suggests that treatment effects on the number and/or severity of OAB symptoms and urodynamic variables may not be fully predictive of treatment effects on HRQL and other PROs that are not based on changes in bladder diary variables, leading some authors to advocate that the use of these PROs become standard in clinical studies of OAB [20].

We evaluated the relationships between treatment-related changes in UUI episodes as recorded in bladder diaries and 2 validated questionnaires using data from a large placebo-controlled trial of tolterodine [21].

## Methods

Details of the study design have been previously published [21]. Briefly, this 12-week, double-blind, placebo-controlled trial included subjects with urinary frequency (≥ 8 micturitions per 24 h) and UUI (≥ 5 episodes per wk) who had OAB symptoms for ≥ 6 months. This study was conducted in 167 centers in Australasia (n = 4), Europe (n = 89), and North America (n = 74). The study was conducted in accordance with the Good Clinical Practice Guidelines and the Declaration of Helsinki. The appropriate International Ethics Committees/Institutional Review Boards approved the study protocol, and all patients gave written informed consent before the start of the study. Subjects were randomized to once-daily treatment with 4 mg tolterodine extended release (ER) or placebo for 12 weeks. For this analysis, only subjects who reported at least some minor bladder-related problems at baseline (PPBC score ≥ 3) were included.

### Symptom assessment using bladder diaries and questionnaires

Subjects completed 7-day bladder diaries in which they recorded every incontinence episode, urgency episode, and micturition at the times at which they occurred during 7 consecutive days prior to the baseline and week 12 visits. Subjects also completed the KHQ and the PPBC at baseline and week 12. The KHQ is a measure of HRQL that includes 2 single-item domains (General Health Perceptions and Incontinence Impact), 7 multi-item domains (Role Limitations, Physical Limitations, Social Limitations, Personal Relationships, Emotions, Sleep/Energy, Severity/Coping), and a multi-item Symptom Severity scale [22]. The 2 single-item domains and the 7 multi-item domains of the KHQ are scored on a scale from 0 (best) to 100 (worst). The Symptom Severity scale is scored from 0 (best) to 30 (worst). Decreases in KHQ domain scores indicate an improvement in HRQL. The minimally important difference (MID) – the smallest change in score that subjects perceive as beneficial – is 3 points for the Symptom Severity scale and 5 points for all other KHQ domains [23].

The PPBC is a validated single-item measure of the subjects' perception of the extent of their bladder-related problems [9,24]. Subjects select 1 of 6 statements ranging from "my bladder condition does not cause me any problems at all" to "my bladder condition causes me many severe problems" [9,24]. A decrease in score indicates improvement.

### Statistical analysis

Median percentage changes in UUI episodes per week were analyzed using a rank analysis of covariance (ANCOVA) with baseline value as a covariate; mean changes in UUI episodes per week were analyzed using ANCOVA with baseline value as a covariate. Mean changes in KHQ scores from baseline to week 12 were analyzed for the current study using ANCOVA with baseline value as a covariate. Categorical changes in PPBC scores were analyzed using chi-square tests. A ≥ 2-point reduction in PPBC score was considered a major improvement, a 1-point reduction was considered a minor improvement, and no change or an increase was considered no improvement in the extent of bladder-related problems. The relationships between changes in UUI episodes, PPBC scores, and KHQ scores were evaluated using Spearman correlation coefficients. Changes in UUI episodes were used for this analysis because they were a defining symptom for this population. Correlations were considered to be small in size for |r| < 0.30 and moderate in size for |r| = 0.30 to 0.49 [25].

## Results

Baseline demographics and clinical characteristics for 987 subjects (placebo, n = 487; tolterodine ER, n = 500) are presented in Table 1. Most subjects were women (82%) and white (95%).

**Table 1:** Baseline demographic and clinical characteristics

**Characteristic**	**Placebo** **(n = 487)**	**TER** **(n = 500)**
Women, n (%)	401 (82)	412 (82)
Age,* y	61 (14)	60 (14)
Range	22–93	20–89
Race, n (%)		
White	460 (95)	477 (95)
Black	19 (4)	17 (3)
Other	8 (2)	6 (1)
UUI episodes per wk*	23.5 (20.7)	22.3 (22.4)
Micturitions per 24 h*	11.3 (3.8)	10.9 (4.2)
Volume voided per micturition,* mL	135.3 (41.9)	140.1 (42.7)

TER = tolterodine extended release; UUI = urgency urinary incontinence.

*Data are mean (SD).

Subjects treated with tolterodine ER showed a significantly greater median percentage (-71% vs -33%, *P *< 0.0001) and mean (-11.8 vs -6.9, *P *< 0.0001) reduction in UUI episodes per week than did subjects treated with placebo. A significantly greater percentage of subjects treated with tolterodine ER showed improvement in PPBC scores (58%; 30% with ≥ 2-point improvement) than did subjects treated with placebo (45%; 21% with ≥ 2-point improvement; *P *< 0.0001). Subjects treated with tolterodine ER also achieved significantly larger improvements on 7 KHQ domains, including Incontinence Impact, Role Limitations, Physical Limitations, Social Limitations, Sleep/Energy, Severity/Coping, and Symptom Severity compared with subjects receiving placebo (Table 2). Among subjects treated with tolterodine ER, the change in every KHQ domain score except Symptom Severity and General Health Perceptions exceeded MID criteria for improvement [23]. The MID criteria were met for the Emotions domain in the tolterodine ER group; however, the improvement from baseline was not statistically significant (*P *= 0.0746) compared with the placebo group.

**Table 2:** Change from baseline to week 12 in KHQ domain scores by treatment group

**KHQ Domain**	**Placebo** **Mean (SD)** **[n]**	**TER** **Mean (SD)** **[n]**	***P *Value**
General Health Perceptions	-0.06 (17.4) [406]	-0.4 (17.6) [419]	0.9850
Incontinence Impact	-8.9 (26.7) [405]	-16.0 (29.4) [417]	<0.0001
Role Limitations	-10.4 (29.3) [404]	-18.1 (30.7) [416]	<0.0001
Physical Limitations	-9.0 (28.1) [404]	-15.8 (30.1) [417]	0.0003
Social Limitations	-6.3 (22.8) [402]	-8.7 (23.4) [411]	0.0431
Personal Relationships	-3.5 (23.2) [265]	-5.8 (27.0) [259]	0.4759
Emotions	-6.4 (24.1) [403]	-9.4 (25.0) [413]	0.0746
Sleep/Energy	-4.8 (21.0) [404]	-9.9 (24.6) [415]	0.0033
Severity/Coping	-6.1 (20.0) [402]	-11.9 (22.1) [414]	<0.0001
Symptom Severity	-1.5 (3.9) [406]	-2.9 (4.1) [419]	<0.0001

KHQ = King's Health Questionnaire; TER = tolterodine extended release.

Among subjects receiving tolterodine ER, the present analysis revealed a moderately sized but statistically significant correlation between week 12 median percentage reductions in UUI episodes and changes in PPBC score (|*r*| ≥ 0.35, *P *≤ 0.001). Small to moderately sized but statistically significant correlations were also observed between median percentage reductions in UUI episodes from baseline and improvements in 8 of 10 KHQ domains (|*r*| = 0.16–0.35, all *P *≤ 0.01) in tolterodine ER-treated subjects (Table 3). Similar correlations were observed between mean reductions in UUI episodes and improvements in PPBC and KHQ scores.

**Table 3:** Correlations between percentage change from baseline in UUI episodes and changes in PPBC and KHQ domain scores in subjects treated with TER or placebo

	**Placebo**		**TER**	
	
	***r***	***P *Value**	***r***	***P *Value**
PPBC	0.32	<0.0001	0.35	<0.0001
KHQ domain				
General Health Perceptions	0.11	0.0252	0.09	0.0687
Incontinence Impact	0.27	<0.0001	0.32	<0.0001
Role Limitations	0.30	<0.0001	0.16	0.0008
Physical Limitations	0.25	<0.0001	0.23	0.0001
Social Limitations	0.13	0.0078	0.16	0.0011
Personal Relationships	0.12	0.0436	0.11	0.0773
Emotions	0.14	0.0041	0.25	<0.0001
Sleep/Energy	0.24	<0.0001	0.19	0.0001
Severity/Coping	0.34	<0.0001	0.30	<0.0001
Symptom Severity	0.33	<0.0001	0.32	<0.0001

KHQ = King's Health Questionnaire; PPBC = Patient Perception of Bladder Condition; TER = tolterodine extended release; UUI = urgency urinary incontinence.

**Figure 1:**
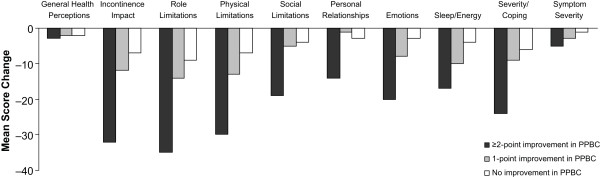
**Mean change in KHQ domain scores by categorical improvement in PPBC scores in TER-treated subjects**. KHQ = King's Health Questionnaire; PPBC = Patient Perception of Bladder Condition; TER = tolterodine extended release.

Treatment-related changes in PPBC scores were correlated with changes in all 10 KHQ domains (Table 4). Similar relationships were observed in the placebo group. Moderate but statistically significant correlations (|*r*| ≥ 0.30, all *P *< 0.001) were observed between PPBC scores and Incontinence Impact, Role Limitations, Physical Limitations, Emotions, Severity/Coping, and Symptom Severity in the tolterodine ER group; the placebo group showed similar correlations except for Emotions and Symptom Severity, which were poorly correlated. Categorical improvement on the PPBC was correlated with improvements in KHQ domain scores in the tolterodine ER group (Figure 1). Changes in KHQ scores were 2 to 3 times larger in subjects who showed a ≥ 2-point improvement on the PPBC than in those who showed a 1-point improvement.

**Table 4:** Correlations between PPBC and KHQ domain change scores in subjects treated with TER or placebo

	**Placebo**		**TER**	
	
**KHQ Domain**	***r***	***P *Value**	***r***	***P *Value**
General Health Perceptions	0.14	0.0038	0.13	0.009
Incontinence Impact	0.41	<0.0001	0.36	<0.0001
Role Limitations	0.31	<0.0001	0.31	<0.0001
Physical Limitations	0.35	<0.0001	0.33	<0.0001
Social Limitations	0.29	<0.0001	0.24	<0.0001
Personal Relationships	0.16	0.0113	0.17	0.0076
Emotions	0.25	<0.0001	0.30	<0.0001
Sleep/Energy	0.22	<0.0001	0.20	<0.0001
Severity/Coping	0.36	<0.0001	0.33	<0.0001
Symptom Severity	0.30	<0.0001	0.38	<0.0001

KHQ = King's Health Questionnaire; PPBC = Patient Perception of Bladder Condition; TER = tolterodine extended release.

There were improvements from baseline in UUI episodes, PPBC, and KHQ in the placebo group that were statistically significantly smaller than those in the tolterodine ER group (Table 2). However, correlations among these endpoints in the placebo group were similar to those observed in the tolterodine ER group (Tables 3 and 4).

## Discussion

In this analysis, the tolterodine ER group showed statistically significant and clinically meaningful improvements in UUI episodes, subjects' perceptions of the extent of their bladder-related problems, and HRQL compared with placebo. These results are consistent with previous reports demonstrating that tolterodine ER significantly improved UUI episodes [26,27], PPBC scores [18,26], and KHQ scores [16,17] in subjects with OAB. However, correlational analyses showed that improvement in UUI episodes was significantly correlated with improvements in PPBC scores and most domains of the KHQ in subjects receiving either tolterodine ER or placebo. The results were similar regardless of whether median percentage or mean changes in UUI episodes were used in the analyses. Changes in PPBC scores were also correlated with changes in scores on each domain of the KHQ, and categorical improvements in PPBC scores were positively associated with the magnitude of improvement on each KHQ domain. These data suggest that the PPBC and KHQ are relevant measures of OAB treatment efficacy.

Although the correlations among improvements in UUI episodes, PPBC scores, and KHQ scores after treatment with tolterodine ER were small to moderate, they were statistically significant. Similarly, a study that used data from primary care patients collected during an open-label trial of tolterodine ER found that improvements in bladder diary variables were significantly correlated with improvements on the PPBC, the Overactive Bladder Questionnaire (OAB-q) Symptom Bother scale, and all HRQL domains of the OAB-q [28]. As in the present study, the correlations were small to moderate but statistically significant. Collectively, these findings suggest that PROs provide information about OAB treatment efficacy that is related to, but not redundant with, bladder diaries. This interpretation is consistent with a study by Abrams et al [20] in which symptom-based outcomes (ie, bladder diaries, urodynamics) were compared with questionnaire outcomes from 27 clinical trials of antimuscarinics. The results showed that, although there were often parallel improvements in these 2 outcomes, there were cases in which improvements in symptom-based measures were not accompanied by corresponding changes in PRO measures.

Abrams et al [20] concluded that statistically significant changes in the frequency of symptoms may not always reflect subjects' assessments of treatment success, and that both symptom-based measures and patient-centric measures of treatment impact should be used in studies of OAB pharmacotherapy [20]. The findings reported here support the conclusion that symptom-based measures and patient-centric measures are complementary and should be used together in clinical trials. The KHQ, PPBC, and other instruments, such as the OAB-q, Overactive Bladder Symptom Score (OABSS) and Urgency Perception Scale (UPS), have been validated among patients with OAB and are reliable and responsive to treatment [9,10,24,29-32].

In the current study, correlations between improvements in PPBC and KHQ scores were also of small to moderate strength, suggesting that these instruments may reflect different aspects of OAB treatment efficacy. The PPBC, a single-item measure that is easy to use and interpret, provides a global integrated measure of the subjects' perception of the extent of their bladder-related problems. Other validated instruments, such as the KHQ or OAB-q, provide detailed and multidimensional information concerning patient outcome or condition that is not captured by a single-item measure. The choice of questionnaires administered in clinical trials should reflect the research objectives of the trial [9].

Notably, these instruments are patient-friendly and may help clinicians diagnose OAB and assess treatment efficacy in the clinic without the inconvenience of a bladder diary. The importance of measuring patients' assessment of treatment impact has been demonstrated by evidence of disparity between the opinions of clinicians and patients regarding the level of bother and the impact of urinary symptoms on HRQL [33].

Although changes from baseline for each endpoint were significantly smaller among placebo- versus tolterodine ER-treated subjects, relationships among endpoints were generally similar in the active and placebo groups. This is not unexpected because the correlations should be similar for changes from baseline that are in the same direction, whether they occur in the treatment or placebo group.

The current analysis suggests that PROs are relevant and complementary to changes in UUI episodes in the assessment of OAB treatment; however, the study has some limitations. Inclusion criteria for this study required subjects to have UUI; therefore, the analysis is limited to only patients with UUI. However, a large proportion of patients with OAB are not incontinent [3-5]. Because UUI was the only variable used to assess these relationships, we cannot extrapolate these results to other OAB symptoms.

## Conclusion

Changes in UUI episodes were correlated with changes in PPBC and HRQL scores in subjects with OAB treated with either tolterodine ER or placebo. These findings indicate that data obtained from PROs provide a distinct source of information about treatment efficacy in the evaluation of OAB treatments.

## Abbreviations

ER: extended release; HRQL: health-related quality of life; ICS: International Continence Society; KHQ: King's Health Questionnaire; MID: minimally important difference; OAB: overactive bladder; PPBC: Patient Perception of Bladder Condition; PRO: patient-reported outcome; TER: tolterodine extended release; UUI: urgency urinary incontinence.

## Competing interests

PVK has been an investigator and lecturer for Astellas, Eli-Lilly, Ferring, Pfizer Inc, and Novartis; CK has been a consultant, investigator, and lecturer for Astellas, GlaxoSmithKline, Novartis, Pfizer Inc, Tanabe, Union Chimique Belge, and Watson; KSC has been a consultant for Pfizer Inc; ZK, MB, and JTW are employees of Pfizer Inc.

## Authors' contributions

PvK, CK, KC, ZK, MB, and JTW participated in the concept and design, interpretation of data, and the drafting and revising of the manuscript. PvK was involved in collecting the data. JTW conducted the data analysis. All authors have read and approved the final manuscript.
